# Genome-wide identification and expression analysis of WRKY transcription factors in pearl millet (*Pennisetum glaucum*) under dehydration and salinity stress

**DOI:** 10.1186/s12864-020-6622-0

**Published:** 2020-03-14

**Authors:** Jeky Chanwala, Suresh Satpati, Anshuman Dixit, Ajay Parida, Mrunmay Kumar Giri, Nrisingha Dey

**Affiliations:** 1 Institute of Life Sciences, NALCO Nagar Road, NALCO Square, Chandrasekharpur, Bhubaneswar, Odisha 751023 India; 2 School of Biotechnology, Campus 11, KIIT (Deemed to be) University, Patia, Bhubaneswar, Odisha 751024 India

**Keywords:** Pearl millet, WRKY transcription factors, Cis-regulatory elements, Synteny, Abiotic stress

## Abstract

**Background:**

Plants have developed various sophisticated mechanisms to cope up with climate extremes and different stress conditions, especially by involving specific transcription factors (TFs). The members of the WRKY TF family are well known for their role in plant development, phytohormone signaling and developing resistance against biotic or abiotic stresses. In this study, we performed a genome-wide screening to identify and analyze the WRKY TFs in pearl millet (*Pennisetum glaucum; PgWRKY*), which is one of the most widely grown cereal crops in the semi-arid regions.

**Results:**

A total number of 97 putative PgWRKY proteins were identified and classified into three major Groups (I-III) based on the presence of WRKY DNA binding domain and zinc-finger motif structures. Members of Group II have been further subdivided into five subgroups (IIa-IIe) based on the phylogenetic analysis. *In-silico* analysis of PgWRKYs revealed the presence of various cis-regulatory elements in their promoter region like ABRE, DRE, ERE, EIRE, Dof, AUXRR, G-box, etc., suggesting their probable involvement in growth, development and stress responses of pearl millet. Chromosomal mapping evidenced uneven distribution of identified 97 *PgWRKY* genes across all the seven chromosomes of pearl millet. Synteny analysis of PgWRKYs established their orthologous and paralogous relationship among the WRKY gene family of *Arabidopsis thaliana, Oryza sativa* and *Setaria italica*. Gene ontology (GO) annotation functionally categorized these PgWRKYs under cellular components, molecular functions and biological processes. Further, the differential expression pattern of *PgWRKY*s was noticed in different tissues (leaf, stem, root) and under both drought and salt stress conditions. The expression pattern of *PgWRKY33*, *PgWRKY62* and *PgWRKY65* indicates their probable involvement in both dehydration and salinity stress responses in pearl millet.

**Conclusion:**

Functional characterization of identified *PgWRKY*s can be useful in delineating their role behind the natural stress tolerance of pearl millet against harsh environmental conditions. Further, these *PgWRKY*s can be employed in genome editing for millet crop improvement.

## Background

Global warming has a substantial impact on sustainability of the crop plants. Agricultural production is becoming more vulnerable due to climate variability [1]. Climate change associated environmental problems such as soil erosion, drought, flood, high temperature and an altered pattern of precipitation results in low and erratic crop yield [2]. Alongside, the increasing human population with intense urbanization affects the crop production and cultivated land area negatively. To ensure future food security, it is an utmost need for promoting the cultivation of major crops along with naturally adapted crops like millets, which can sustain under harsh environmental conditions [3].

Pearl millet (*Pennisetum glaucum*), syn. *Cenchrus americanus*, is one of the most widely grown crop in the arid and semi-arid tropical regions of Africa and South-east Asia including India. It serves as one of the staple food for millions of poor people and is also being used extensively for fodder and fuel [4]. It is highly resilient and well adapted to severe abiotic stresses including elevated temperature, drought and high soil pH. A mean annual rainfall of around 250–300 mm is sufficient for pearl millet grain production, where most of the other important crops like rice, wheat, sorghum and maize are likely to fail [5]. Apart from this advantage of growing in adverse environmental conditions, pearl millet also has high nutritional index compared to rice, wheat, sorghum and maize. Pearl millet contains 8–19% protein, low starch, high fiber and essential micronutrients such as iron and zinc [6, 7]. Due to these characteristics, worldwide attention is now focused on pearl millet cultivation to cope up with climate change and food insecurity [8].

Abiotic stresses cause damages to crop productivity and it accounts for more than 50% agricultural production losses. Drought and salinity are two major constraints having a multidimensional impact on growth and productivity of the crops as they result in depleted groundwater tables, photosynthetic inhibition, reduced membrane protein stability and changes physiochemical properties of soil [9]. It has been seen that a 10% drop in rainfall results in an average of 4.2% decrease in cereals yield [10]. All water constraints, including drought results in 15–30% of agricultural yield losses [11]. Likewise, salinity also drastically affect crop productivity. On average, higher than normal salinity conditions prevail in 20% of cultivated and 33% of irrigated land globally [12]. All-important glycophytic crop plants reduce their average global yield by 50–80% under moderate salinity conditions [13, 14].

Plants have adapted several ways to escape such environmental stresses by employing several integrated transcriptional and hormonal factors. Specific transcription factors (TFs, the regulatory proteins) bind to the respective cognate cis-elements present in the promoter region of their target genes and modulate the expression level of genes under particular stress conditions. Such “cis-trans” interactions manifest significantly for controlling the plant survival under adverse environmental conditions [15]. In plants, several TF families have been reported namely ABRE-binding factor (ABF)/ABA-responsive-element-binding (AREB) [16], ethylene responsive element binding factors (ERF) [16], DREB [17], NAC [18], AP2/ERF [19], WRKY [20], MYB [21], MYC [22] and basic domain leucine zipper (bZIP) [23] etc.

Structurally, WRKY transcription factors have conserved WRKY domain with signature sequence (WRKYGQK) along with zinc-finger motif (C-C, H-H/C) [24]. Broadly, WRKY transcription factors are classified into three major groups based on the number of WRKY domains and arrangement of the zinc-finger motif. Group I protein sequences contain two WRKY domains (at both N and C terminal) along with C_2_H_2_ zinc-finger motif (CX_4-5_C X_22-23_HXH). Group II proteins have only one WRKY domain followed by a C_2_H_2_ zinc-finger motif (CX_4-5_CX_22-23_HXH). Further, Group II proteins are classified into five subgroups, namely IIa, IIb, IIc, IId and IIe based on sequence characteristics and phylogenetic analysis. Like Group II proteins, Group III proteins also have a WRKY domain. However, instead of C_2_H_2_ motif, a C_2_HC zinc-finger motif (CX_7_CX_23_HXC) is conserved in Group III members [24–26]. Recent studies have assigned WRKY proteins possessing WRKY domain with no or partial zinc-finger motif structure to a separate group (Group IV; uncharacterized) [27–31].

Considering that WRKY TF is one of the key biological regulators, several studies have characterized their role in various plant species like foxtail millet, wheat, cotton and grapevine etc. [28–30, 32–36]. However, no such studies have been reported that may provide extensive insights about the role of WRKY TFs in pearl millet (*P. glaucum*). In this study, we have undertaken approaches for genome-wide identification of putative WRKY proteins present in pearl millet, their classification into different groups, chromosomal distribution, presence of conserved motifs, phylogenetic relationship, and sequence homology with WRKY family members of *Arabidopsis thaliana*, *Oryza sativa* (rice), and *Setaria italica* (foxtail millet). Further, we analyzed the relative expression profile of *WRKY* genes in different plant tissues and in response to drought and salinity stresses. The findings of this study will facilitate us to understand the mechanism behind the natural adaptation of pearl millet under abiotic stress. Also, candidate pearl millet *WRKY* genes can be employed in designing genetically improved millet for boosting agricultural production.

## Results

### Identification of the WRKY transcription factors in *P. glaucum*

The HMMSCAN search resulted in the identification of 97 WRKY (PgWRKY1 to PgWRKY97) transcription factors from the complete proteome database of *P. glaucum*. Further, protein sequence length, molecular weight (MW), isoelectric point (pI) and other indexes were analyzed for all identified 97 PgWRKYs of *P. glaucum*. We observed that the sequence length of the WRKY proteins varies from 123 amino acids (PgWRKY16) to 1394 amino acid residues (PgWRKY85). Their MW ranges from 13.732 to 156.285 kDa, and the pI ranges from 4.49 to 10.29 (Additional file 1).

### Classification of PgWRKY proteins and phylogenetic analysis

**Fig. 1:**
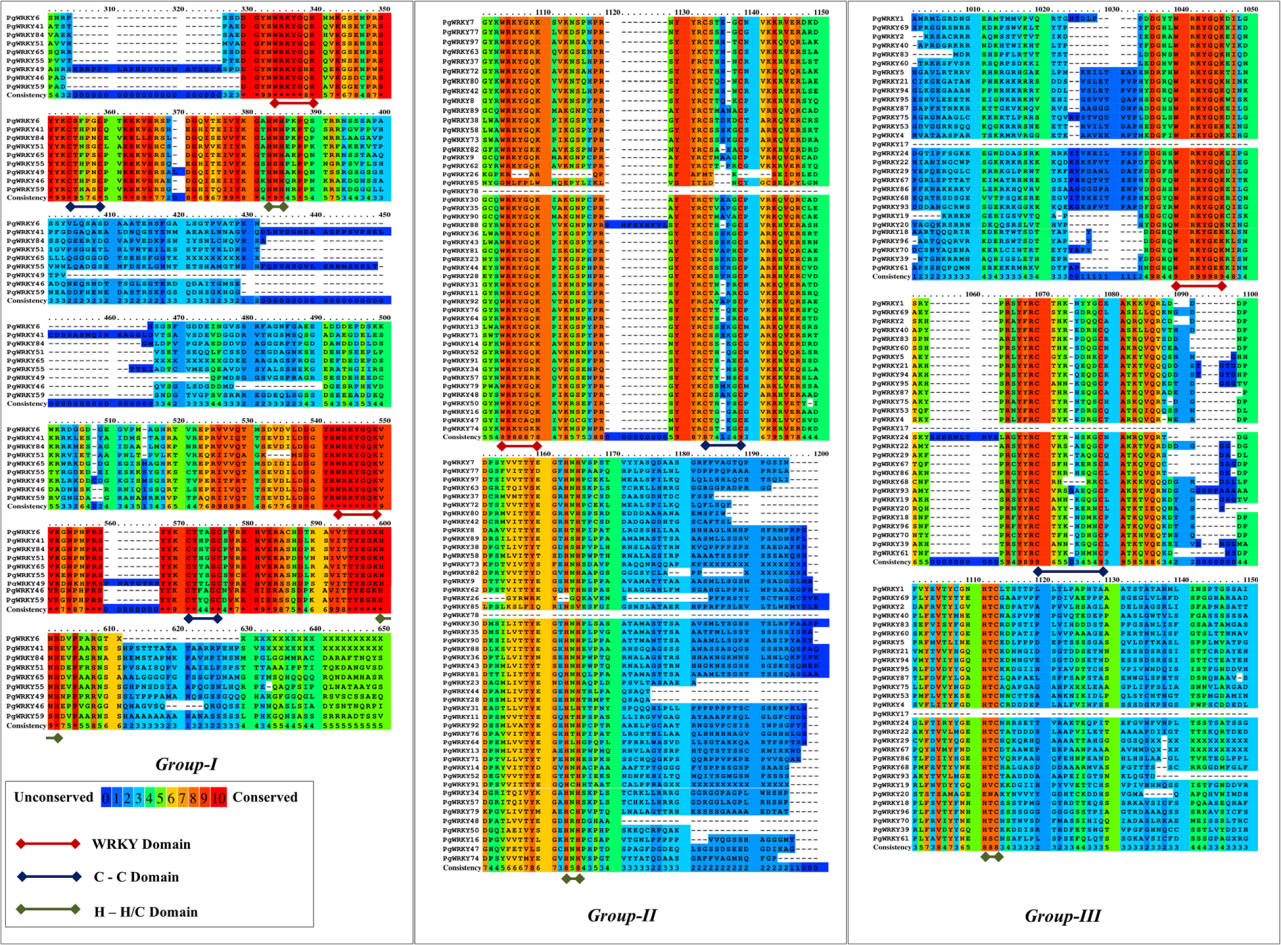
Multiple sequence alignment of identified PgWRKY proteins. The amino acid conservation is shown in shaded colours, while domain conservation is shown through underline colours. The shaded colours indicate low to high residue conservation i.e., blue to red. The domain conservation for WRKY, C-C and H-H/C domains are shown through underline red, blue and green colour respectively

The PgWRKY proteins were examined for conservation of the WRKY domain using multiple sequence alignment. As shown in Fig. 1, the sequences with amino acid conservation were shown in blue to red colour index where blue indicates the least and red means highly conserved patches. Multiple sequence alignment showed high conservation of “WRKYGQK” motif and “zinc-finger motif” in all identified PgWRKYs. Identified 97 PgWRKY proteins were classified into three groups based on the number of WRKY domains and structure of zinc-finger motif. Among the identified 97 PgWRKYs, we observed 9 PgWRKYs belongs to Group I; 47 PgWRKYs belong to Group II (forming the largest group); 29 PgWRKYs belong to Group III. Furthermore, we did not observe an intact zinc-finger motif in remaining 12 PgWRKYs. This is consistent with earlier studies conducted on *Setaria italica*, *Gossypium hirsutum* and *Musa balbisiana* [28, 29, 32]*.* Hence, these 12 PgWRKYs were kept in a separate group (Group IV; uncharacterized). Most of the PgWRKYs contain the conserved “WRKYGQK” motif, whereas few PgWRKYs have slight variations in their signature motif (Additional file 2).

**Fig. 2:**
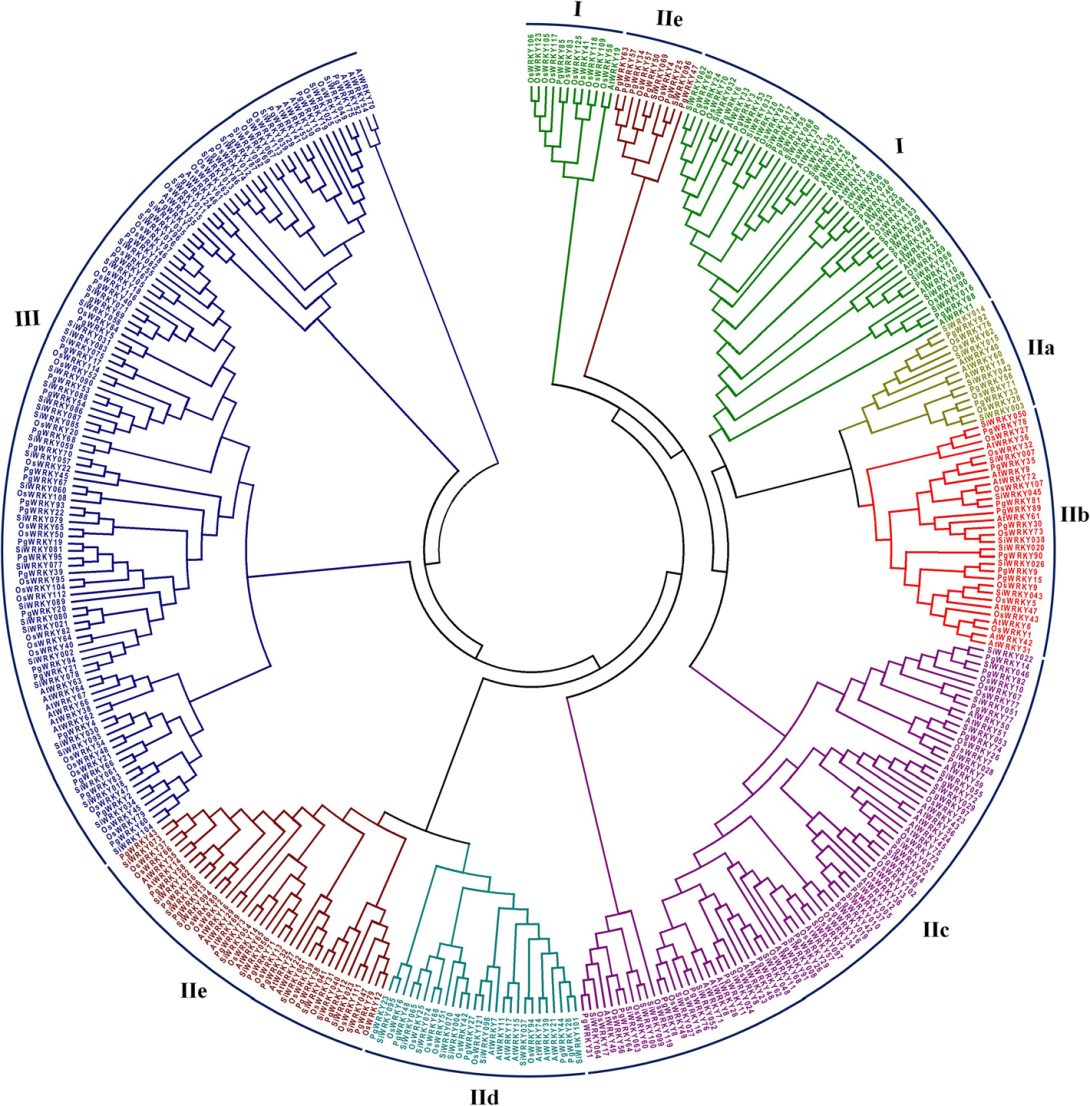
The circular phylogenetic representation of *P. glaucum* WRKY proteins with *A. thaliana, O. sativa* & *S. italica:* A total of 379 WRKY proteins were aligned by MUSCLE, and a phylogenetic tree was constructed by MEGA v7.0 using maximum likelihood method with 1000 bootstrap replication. Each colour indicates an individual group (I-III) of ancestral relationship

A phylogenetic study was performed to analyze the evolutionary relationships among the WRKY families of *A. thaliana, O. sativa, S. italica* and *P. glaucum*. A total of 379 WRKY proteins including 72 from *A. thaliana*, 105 from *O. sativa*, 105 from *S. italica*, and 97 from *P. glaucum* were used to construct a phylogenetic tree as described in the method section. As shown in Fig. 2, all 379 WRKYs were clustered across major clades. We observed WRKY members belonging to a specific group (I, II, III) of all analyzed species were also clustering to the same clade (highlighted in Fig. 2).

### Chromosomal distribution and structure analysis of *PgWRKY* genes

**Fig. 3:**
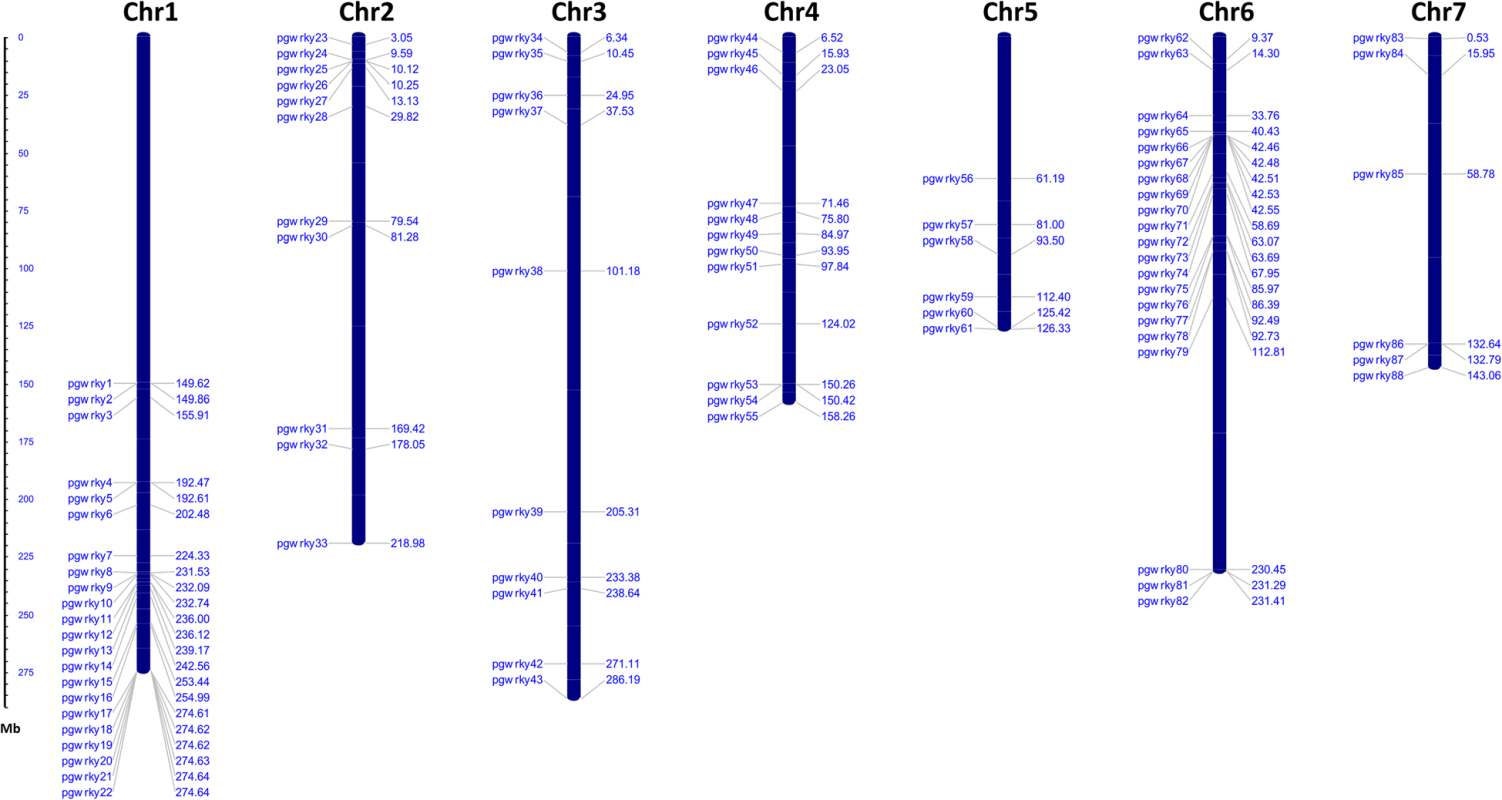
The chromosomal distribution and positioning of *PgWRKYs* across all seven chromosomes of *P. glaucum.* Seven chromosomes with varying lengths are shown in Mb (million base pair) scale in the left, where individual chromosomes (bars) are labelled with respective *PgWRKY* genes

Identified *PgWRKYs* were mapped on seven chromosomes of *P. glaucum* (Fig. 3). Eighty-eight *PgWRKYs* were unevenly distributed across the *P. glaucum* genome. Remaining 9 *PgWRKYs* were not mapped due to unavailability of chromosomal coordinates in the genome database. Most of the *PgWRKYs* were abundant on 1st (22 genes; ~ 23%) and 6th (21 genes; ~ 22%) chromosomes whereas least were found on 5th and 7th (6 genes each; ~ 6%) chromosomes. A total number of 19 *PgWRKYs* were located at the telomere region of chromosome 1, while 17 *PgWRKYs* were traced at the centromere region of chromosome 6. *WRKY* members of all groups were present on all chromosomes except chromosome 2 and 3, where Group I and IV members were not present respectively (Additional file 3; Figure S1).

**Fig. 4:**
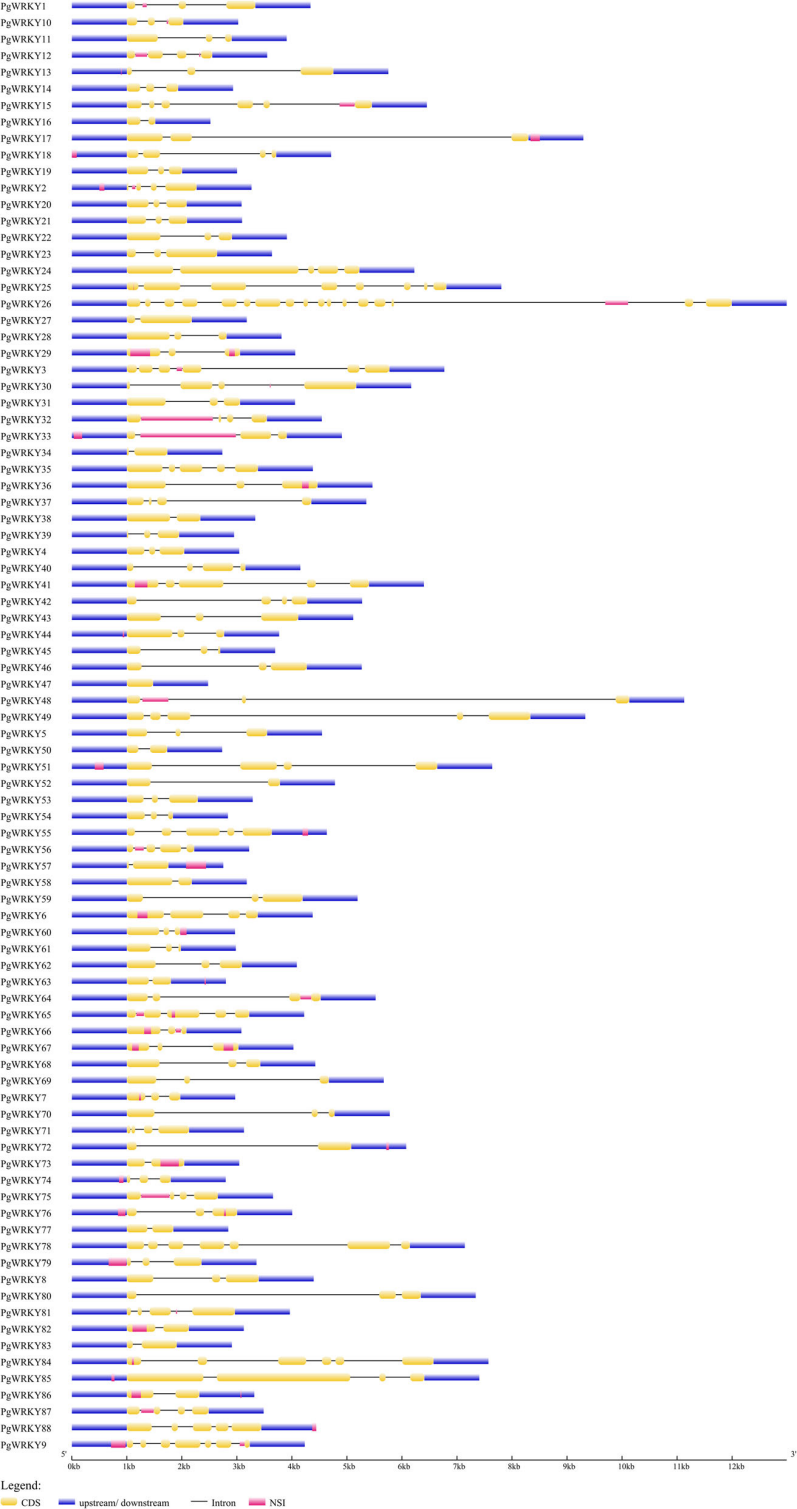
Structural elucidation of identified 97 *PgWRKY* genes: The structural features of *PgWRKYs* are represented in different colours, where yellow indicates an exonic region, blue indicates upstream/downstream region, black indicates intronic region and pink indicates no sequence information

The structural features of identified *PgWRKY* genes were examined in detail using the GSDS server. Figure 4 showed the varying pattern of total exonic and intronic regions in identified 97 *PgWRKYs*. Among 88 *PgWRKYs*, the majority of *PgWRKY* genes (46.59%) had two introns and three exons; followed by 15 *PgWRKYs* with one intron and two exons;17 *PgWRKYs* with three introns and four exons; 7 *PgWRKYs* with four introns and five exons; 3 *PgWRKYs* with five introns and six exons; 2 *PgWRKYs* with six introns and seven exons; 1 *PgWRKY* with seven introns and eight exons; 1 *PgWRKY* with sixteen introns and seventeen exons. However, *PgWRKY47* had no introns (Additional file 1). We also observed variation in gene size of identified *PgWRKYs*, which was ranging from 476 bp (*PgWRKY47*) to 10,991 bp (*PgWRKY26*).

**Fig. 5:**
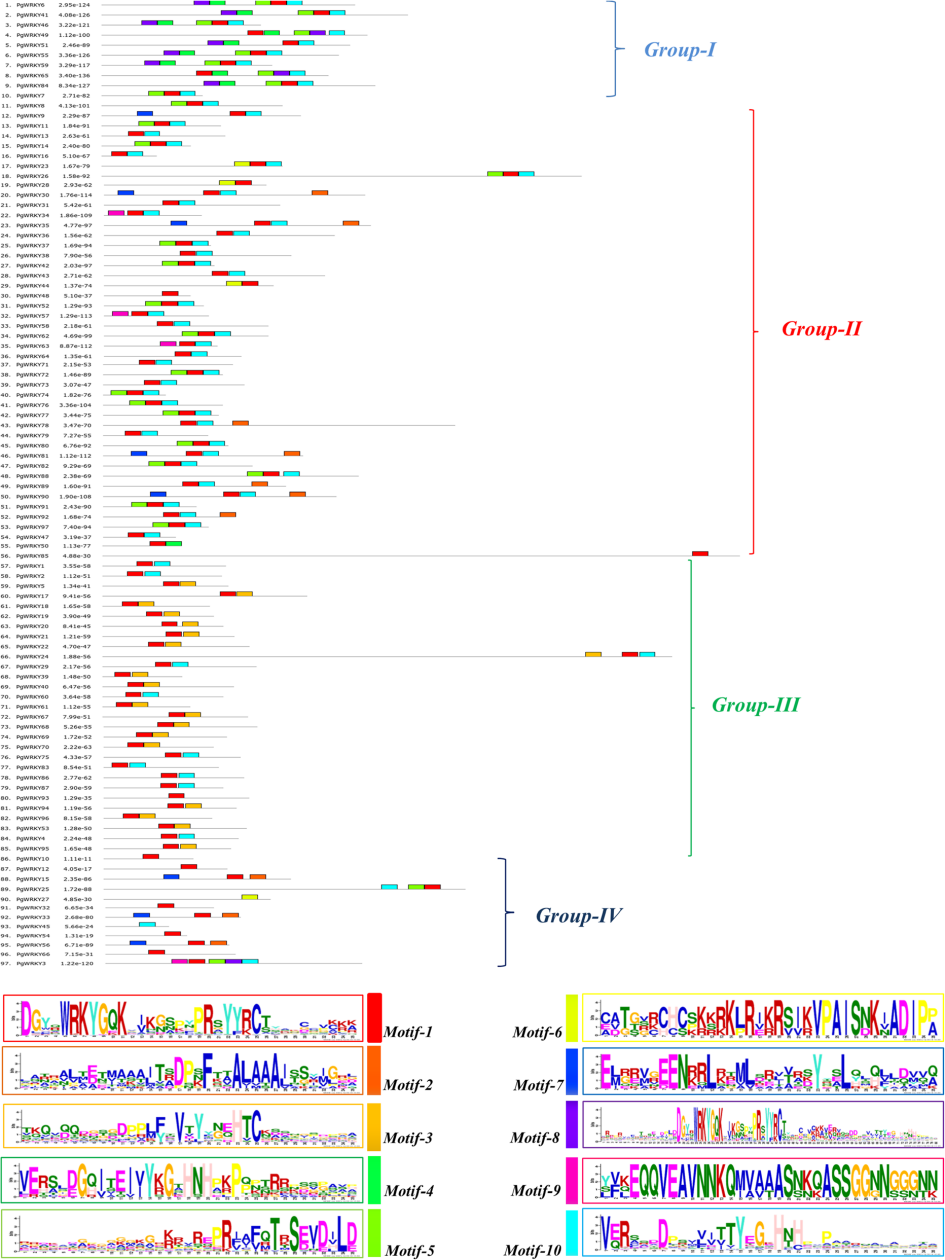
The schematic representation of motif analysis: The upper panel indicates predicted motifs in PgWRKYs, represented in different colour using MEME suite v5.1.0. Whereas, the lower panel shows the signature of each motif with conserved amino acid residues

Further, the motif analysis was performed to identify the conserved motifs present in PgWRKYs using the MEME suite. Schematic presentation of motifs (Fig. 5) revealed that PgWRKYs contain different types of conserved motifs. We identified ten conserved motifs and named them as motif 1 to motif 10 in 97 PgWRKYs. Motif 1 (WRKY motif) was widely distributed in all members of PgWRKY family and motif 8 (WRKY motif) was only present in Group I members. We also observed group-wise specific motif conservation, i.e., motif 4 was found only in Group I members. Similarly, motif 3 was found to be present only in Group III members. We observed Group II members have a different motif distribution pattern according to subgroups (IIa-IIe), such as motif 2 was specific in Group IIa and IIb; motif 7 in Group IIb; motif 5 in Group IIc and motif 6 in Group IId members. We did not find any conserved motif in Group IIe. Group IV members did not possess any specific motif; however, motif 2, motif 7 and motif 5 were partially conserved in few members of Group IV (Additional file 4).

### Synteny relationship and selection pressure analysis of *WRKY* orthologous genes

**Fig. 6:**
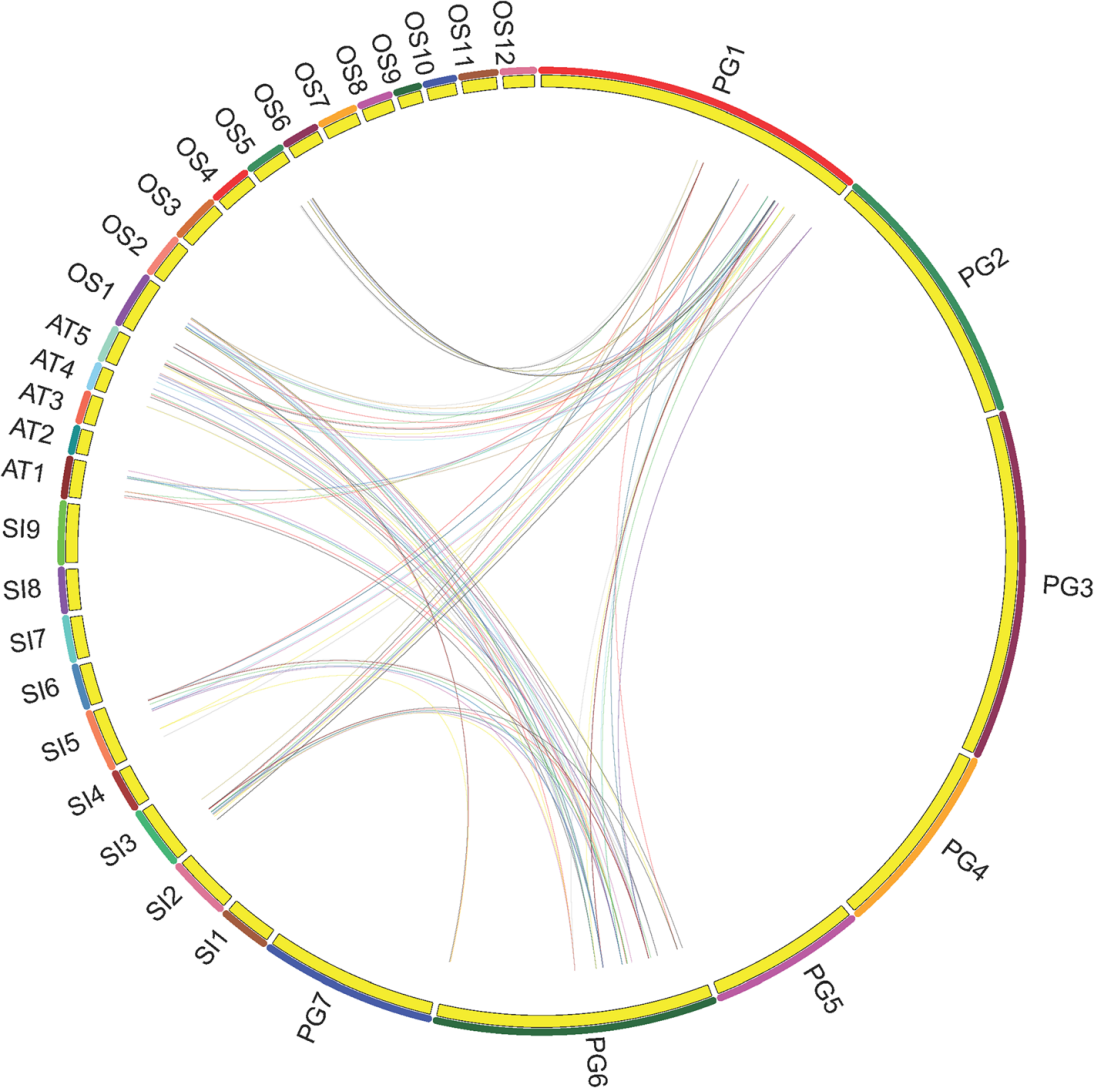
The Circos plot representation for synteny relationship between *P. glaucum*, *A. thaliana, O. sativa* and *S. italica* WRKY genes: The synteny analysis of PgWRKYs were mapped in a circular form using CIRCOS v0.52. Each coloured curve indicates the orthologous positions of PgWRKYs across the chromosomes

Additionally, we attempted to identify the duplication event and analyzed the synteny relationship among the WRKYs of *P. glaucum*, *A. thaliana*, *O. sativa* and *S. italica.* A total number of 33 chromosomes (*P. glaucum*– 7, *A. thaliana*- 5, *O. sativa-*12, *S. italica*– 9) with a total number of 370 WRKYs (*P. glaucum*– 88, *A. thaliana*- 72, *O. sativa-*105, *S. italica*– 105) were used to map the synteny relationships. In Fig. 6, the WRKYs that were involved in segmental duplication and orthologous events were presented by different coloured lines. PgWRKYs from Chromosome 1 (PG1) and Chromosome 6 (PG6) having orthologous pairs with AT1, AT4, AT5 (*A. thaliana*); SI3, SI5 (*S. italica*) and OS1, OS5 (*O. sativa*) chromosomes, indicating hot-spots of PgWRKYs distribution. A total number of 10 pairs were tandemly duplicated and 13 pairs were segmentally duplicated (Additional file 5). We found 97 orthologous pairs of PgWRKYs among WRKYs of *A. thaliana*, *O. sativa* and *S. italica* (Additional file 6). The Ks/Ka ratio of all identified collinear pairs was less than 1, indicating synonymous substitution or purifying selection of PgWRKYs during evolution (Additional file 7).

### Gene ontology annotation and cis-regulatory elements analysis

**Fig. 7:**
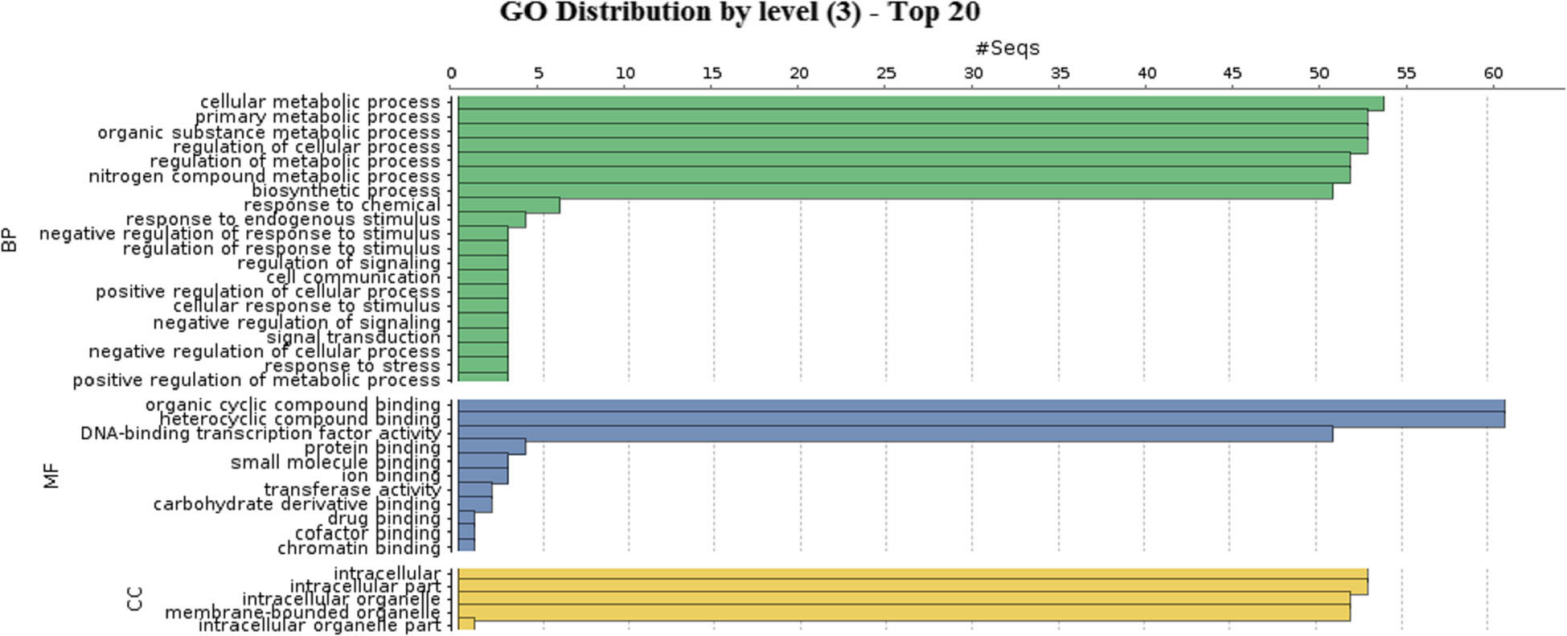
Gene ontology analysis of identified PgWRKYs: The enrichment analysis of PgWRKY shows significantly enriched GO terms involved in cellular, molecular and biological processes

**Fig. 8:**
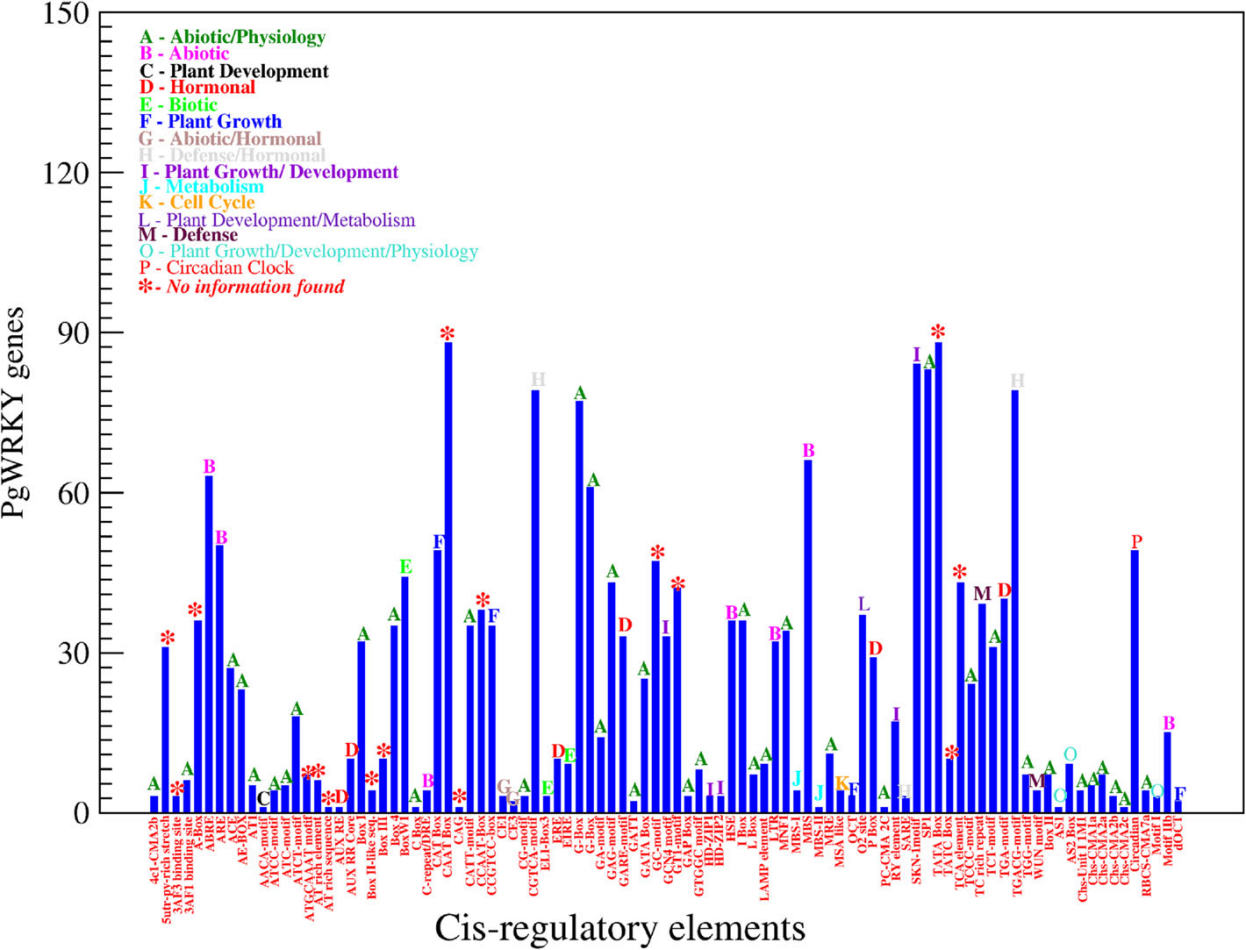
*In-silico* analysis of Cis-regulatory elements: The frequency of identified putative cis-acting elements in the 1.5 kb upstream region of *PgWRKY* genes. The letter on each bar indicates the class (shown in legend) it belongs

Gene ontology (GO) annotations of 97 PgWRKY proteins were predicted using protein blast in Blast2GO tool. Based on identified GO terms, the involvement of identified PgWRKY proteins in various biological processes, cellular components and molecular functions were shown in Fig. 7. A majority of biological processes were predicted to be involved in different metabolic pathways and response to stress conditions. The molecular functions of these proteins predicted to be involved in sequence specific DNA-binding transcriptional activity. The promoter analysis of *PgWRKY* genes was done using PlantCARE database by taking 1.5 kb upstream region. A total number of 127 cis-regulatory elements (CREs) were identified among *PgWRKY* genes (Additional file 8). These cis-elements in *PgWRKY*s were found to be specific to abiotic stress (ABRE, ARE, DRE, HSE, LTR, MBS, ACE, AE-Box, MNF1, MRE, SP1, etc.); biotic stress (EIRE, ELI-Box 3, BoxW1, WUN motif); hormonal; physiological and plant development process (AUX RR, CE1/3, GCN4 motif, SARE, MBS-I/II, MSA like, SKN motif, AS1/2, dOCT). Presence of such versatile cis-elements reflecting the functional divergence of *PgWRKYs* in *P. glaucum* (Fig. 8).

### Relative expression analysis of *PgWRKYs*

WRKY transcription factors are well-known for their regulatory function in various stress signaling pathways. Twenty-five *PgWRKY* genes were selected based on their sequence similarity, blast analysis, motif conservation, synteny and phylogenetic relationship with well-characterized WRKY genes of other species that are shown to be involved in abiotic stress tolerance (Additional file 9). These selected genes were subjected to transcript abundance analysis using qRT-PCR to check their relative expression in different tissues and their probable involvement in dehydration and salinity stresses. PCR conditions for these *PgWRKY* genes were standardized by using PgWRKY specific primers (Additional file 10) with genomic DNA of pearl millet as a template (Additional file 11; Figure S2).

**Fig. 9:**
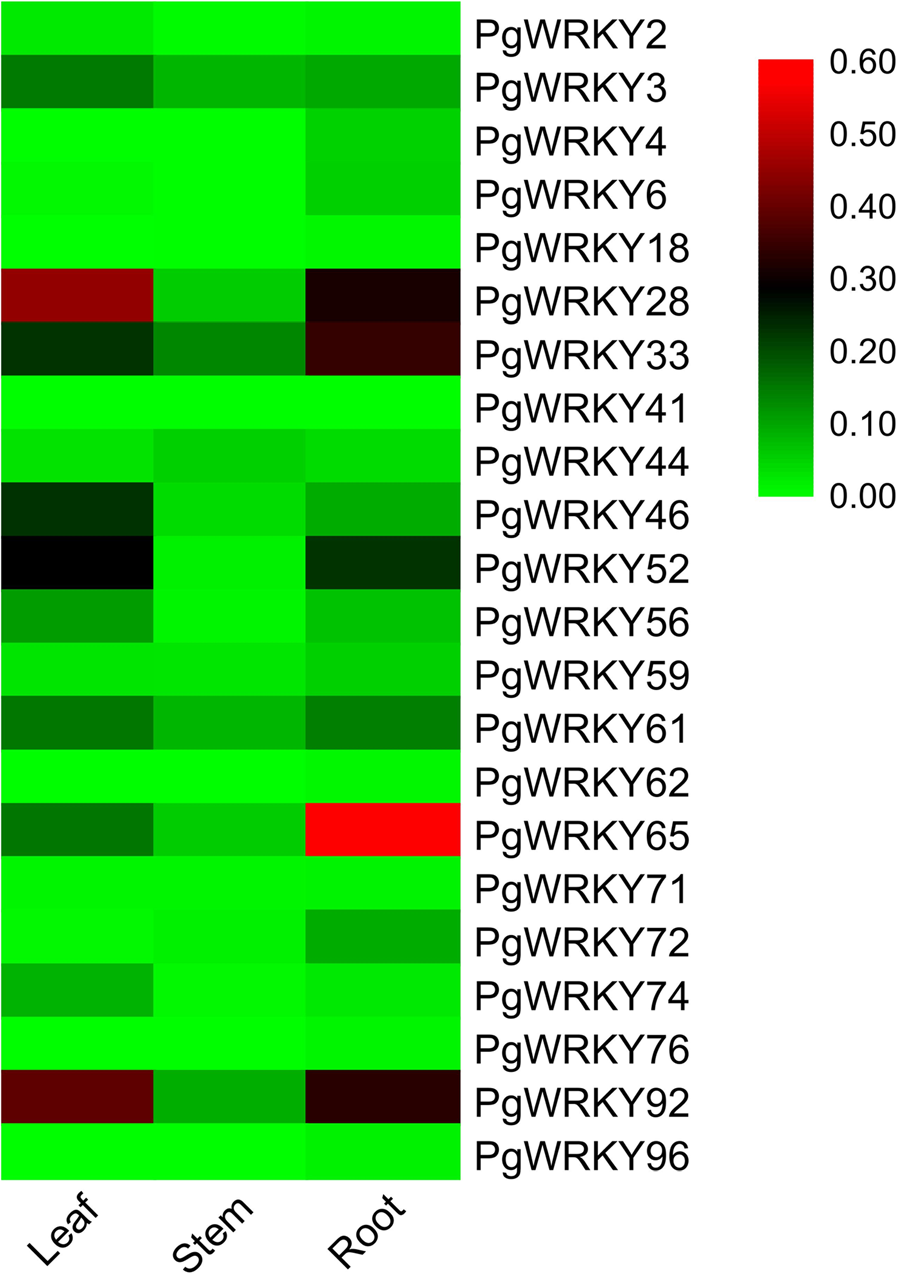
Tissue specific expression analysis. Heat map showing the differential expression level of selected *PgWRKY*s in leaves, stem and root tissues of pearl millet. The transcript abundance level has been normalized and hierarchically clustered. Block with colours indicate decreased (green) or increased (red) transcript accumulation among the analyzed tissues. The heat map was generated using TBtools v0.66831 [37]

Tissue specific expression analysis was performed in leaf, stem and root tissues of pearl millet. Expression analysis showed that 22 *PgWRKYs* were expressed in at least one of the selected tissues (Fig. 9, Additional file 12; Figure S3). While *PgWRKY16, PgWRKY39* and *PgWRKY55* were not expressed in any of the analyzed tissues. *PgWRKY4, PgWRKY18* and *PgWRKY96* were predominantly expressed only in root tissues. Moreover, the majority of the *PgWRKYs* were showing less expression in stem compared to leaf and root tissues, except *PgWRKY41* and *PgWRKY44.* Additionally, 8 *PgWRKYs* (*PgWRKY*2, *PgWRKY3, PgWRKY28, PgWRKY46, PgWRKY52, PgWRKY56, PgWRKY74* and *PgWRKY92*) showed relatively higher expression in leaves compared to stem and root; 10 *PgWRKYs (PgWRKY4, PgWRKY6, PgWRKY18, PgWRKY33, PgWRKY59, PgWRKY62, PgWRKY65, PgWRKY72, PgWRKY76* and *PgWRKY96)* showed relatively higher expression in roots compared to leaf and stem; 2 *PgWRKYs (PgWRKY41* and *PgWRKY44)* showed relatively higher expression in stem compared to leaf and root tissues, while remaining 2 *PgWRKYs* showed similar expression pattern.

**Fig. 10:**
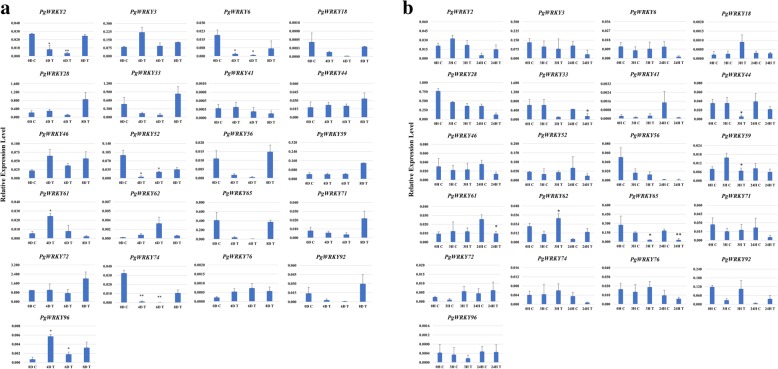
Expression analysis of *PgWRKYs* under different abiotic stress conditions determined by qRT-PCR. The Y-axis indicates relative expression level and the X-axis represents different time points of stress treatment taken for expression analysis. a). Differential expression level of *PgWRKYs* in response to drought stress at 0th day, 4th day, 6th day and 8th day time points. b). Expression pattern of *PgWRKYs* under salt stress at 0-h, 3-h and 24-h time points. Each data point represents mean ± standard deviation (SD) (*n* = 3). Significant difference in mean between the samples for a given set is indicated by *P < 0.05, **P < 0.01, as obtained by Student’s t-test

The expression patterns of selected PgWRKYs were analyzed under drought and salt stress conditions at different time points using qRT-PCR. As shown in Fig. 10a and b, most of the *PgWRKYs* showed differential expression levels under both dehydration and salinity stress conditions at different time points. Specifically, under drought stress condition, we observed the upregulation of six *PgWRKYs* and downregulation of nine *PgWRKYs* in terms of their transcript abundance. We found the expression level of *PgWRKY96* and *PgWRKY61* were significantly induced under drought stress condition. Whereas *PgWRKY2, PgWRKY6, PgWRKY52,* and *PgWRKY74* were significantly downregulated. Similarly, under salt stress condition five *PgWRKYs* were upregulated and nine *PgWRKYs* were down regulated when compared to control samples. The salt treated plants showed significant upregulation of *PgWRKY62* and downregulation of *PgWRKY33, PgWRKY44, PgWRKY59, PgWRKY61* and *PgWRKY65,* compared to control plants at respective time points. The transcript abundance profile of *PgWRKY62* showed similar upregulation pattern in both drought and salt stress conditions. Likewise, the expression pattern of *PgWRKY33* and *PgWRKY65* was found to be downregulated in both drought and salt stress conditions. In addition, we could not detect the transcripts of *PgWRKY4*, *PgWRKY16, PgWRKY39* and *PgWRKY55* in both semi-quantitative as well as in quantitative RT-PCR analysis under drought and salt stress treatments.

## Discussion

Pearl millet (*Pennisetum glaucum*) is an important member of the C4 grass family and it is cultivated in marginal lands with inadequate irrigation and resources. Regardless of its importance as a promising crop, its genomic resources like transcription factor families that play vital roles in gene regulation under stress conditions are not well studied yet. Among the plant TFs families, WRKY TFs family is one of the largest and important family with a broad range of functions in controlling plant growth, development, signal transduction and stress responses. Owing to its importance, the genome-wide identification of WRKY TFs has been carried out in pearl millet.

In this study, we have reported a total number of 97 PgWRKYs *(PgWRKY1 to PgWRKY97)* by complete genome screening of *P. glaucum.* These were categorized into different groups (I, II, III, IV) and subgroups (IIa, IIb, IIc, IId, IIe) based on the presence of conserved WRKY domain and zinc-finger motif like structure. Group IV members are devoid of an intact zinc-finger motif. We assume the loss of zinc-finger motif could modify the functional properties of these *WRKY* genes. However, it is still unclear whether the absence and variants of this zinc-finger motif affect the function and expression of *WRKY* genes [38]. In the course of evolution, variability in the N terminal domain sequence leads to the generation of new WRKY structures or different iso-forms of WRKYs [36]. The WRKY signature motif variants were reported in many crop plants like rice, wheat, maize, soybean and watermelon [38] etc. Similarly, we also found substitutions and variations in amino acid residues of the signature motif of PgWRKYs. Such variants of WRKY motif can amend DNA binding activity that could lead to functional diversification of *WRKY* genes [39]. In this context, the functional binding specificities of these PgWRKYs can be investigated extensively as future prospects of this study.

Phylogenetic analysis and clade formation support group-wise (I, II and III) classification of PgWRKYs. The close phylogenetic relationship was shown by subgroups IIa and IIb, likewise IId and IIe. It suggests the evolution of these subgroups occurred collaterally from a common ancestor. Interestingly, 12 PgWRKYs from an uncharacterized group (Group IV) were sharing clade with Group I, II and III members. This signifies that Group IV members could be evolved from these three group (I, II and III) members in the course of evolution. For example, PgWRKY3 (a Group IV member) has two WRKY domains with no zinc-finger motif at N-terminal that clustered with Group I members. It clearly suggests that it may be evolved from Group I WRKY family of *P. glaucum* [39, 40]. The phylogenetic tree analysis, based on sequence similarity with functionally characterized WRKYs of *A. thaliana, O. sativa* and *S. italica* allowed for predicting and conferring the possible involvement of PgWRKYs in stress responsive processes.

The differential distribution pattern of *PgWRKYs* across the pearl millet genome implies that some chromosomal rearrangements and duplication events might have taken place in the course of evolution. The considerable variation in size, number of introns and exons in *PgWRKYs* suggests the loss and gain of genomic regions occurred during expansion of *WRKY* gene family [41]. In continuation, motif conservation analysis revealed the abundance of different type of motifs in various group members of WRKY family. Predominantly, the WRKY motif was present in all PgWRKYs, whereas some additional specific structural motifs were conserved in few PgWRKY sequences. Such structural stress-specific motifs may manifest functional specificity of these PgWRKYs under various abiotic and biotic stress conditions.

Gene duplication events are significant in the expansion and evolutionary progress of gene families [42]. Comparative mapping established the orthologous and paralogous relationship (Synteny) among dicotyledonous (Arabidopsis), and monocotyledonous (Rice, foxtail millet, pearl millet) plants. Thirty-Six PgWRKYs (~ 41%) were exhibited the synteny relationship with *A. thaliana, O. sativa* and *S. italica*. Interestingly, the presence of 13 collinear pairs between *P. glaucum* and *S. italica*, 14 collinear pairs between *P. glaucum* and *O. sativa* but not in between *P. glaucum* and *A. thaliana*, may suggest that these orthologous pairs were formed after the divergence of dicot and monocot plants. Similarly, the existence of 10 collinear pairs between *P. glaucum* and *A. thaliana* but not in between *P. glaucum, O. sativa* and *S. italica*, may suggest that these orthologous pairs may be involved in divergence of dicot and monocot plants. Additionally, 11 PgWRKYs were collinearly paired with WRKYs of *A. thaliana, O. sativa* and *S. italica,* which indicates that these orthologous pairs may already exist before the ancestral divergence [43]. The functional information of PgWRKYs can be predicted according to their identified orthologous WRKYs in Arabidopsis, Rice and Foxtail millet. Synteny relationship indicates that some *PgWRKY* genes were possibly generated by gene duplication and rearrangement events. The Ks/Ka ratio of identified collinear pairs emphasizes more on negative pressure selection according to Darwin’s natural selection process for amino acid conservation. The comparison study revealed that there was a higher number of orthologs pair with respect to paralogs. This shows that orthologs evolve with a higher evolutionary rate unlike paralogs [44].

Identified *PgWRKYs* conserved with various types of cis-regulatory elements in their promoter regions, suggesting that these *PgWRKYs* could be involved in different biological processes associated with plant growth and development [32]. The presence of various abiotic stress-specific cis-regulatory elements (Fig. 8) in the promoter region of identified *PgWRKYs* may be associated in providing natural tolerance to *P. glaucum.* Furthermore, the presence of various cis-regulatory elements responding to phytohormones (ABA, MeJA and SA), indicate their involvement in controlling various hormonal signaling pathways linked with abiotic and biotic stress management in *P. glaucum*.

Selected twenty-five *PgWRKYs* for expression analysis were representing all four groups (I, II, III, IV) and more or less evenly distributed throughout the genome except chromosome 7 of *P. glaucum.* These *PgWRKYs* showed differential expression levels in leaf, stem and root tissues. Most of the selected *PgWRKYs* showed higher expression in leaves and root as compared to the stem tissues. *PgWRKY4, PgWRKY18* and *PgWRKY96* were found to be expressed predominantly in root tissue. Similarly, transcripts of *PgWRKY2, PgWRKY46* and *PgWRKY74* were most abundant in leaf compared to root and stem tissues. This suggests that these *PgWRKYs* might be related to tissue specific development and signaling processes in *P. glaucum.* Interestingly, transcripts of *PgWRKY16*, *PgWRKY39* and *PgWRKY55* (belonging to Group III) could not be detected in any of these tissues; implying that these *PgWRKYs* could be involved in other physiological processes of *P. glaucum*. Previous studies also indicated that WRKY genes participate in transcriptional regulation of downstream target genes that are involved in various physiological and developmental pathways [20, 25].

The WRKY gene family is significantly involved in regulation of plant responses to various abiotic and biotic stresses [20, 45–48]. *PgWRKYs* exhibited differential expression patterns at different time points of dehydration and salinity stress treatment. For instance, P*gWRKY3, PgWRKY46, PgWRKY61* and *PgWRKY96* were upregulated while *PgWRKY52* was downregulated at 4th day time point under dehydration stress. Similarly, in salt stress condition, transcripts of *PgWRKY18* and *PgWRKY72* were enhanced after 3 h (early time point) and *PgWRKY*2 at 24 h (late time point) post treatment. In addition, few *PgWRKYs* were remarkably suppressed, including *PgWRKY44* and *PgWRKY59* at early time point and *PgWRKY3, PgWRKY6, PgWRKY46, PgWRKY61 and PgWRKY74* at late time point of NaCl treatment. Such differential expression of *PgWRKYs* under stress conditions at different time points suggest their intricate response/s in the regulatory network of plant abiotic stress processes.

The *PgWRKY2*, *PgWRKY6*, *PgWRKY61*, *PgWRKY62, PgWRKY65* and *PgWRKY74* were significantly expressed under dehydration stress. Interestingly, the transcript abundance of these genes on 8th day (after recovery) post treatment was comparable with zero-day (control) samples, which strongly indicates the involvement of these *PgWRKYs* in drought stress response (Additional files 13 and 14; Figure S4). The expression pattern PgWRKY62, *PgWRKY33*, *PgWRKY44*, *PgWRKY59*, *PgWRKY61* and *PgWRKY65* indicated their involvement in salinity stress responses of pearl millet.

The expression patterns of *PgWRKY62*, *PgWRKY33* and *PgWRKY65* suggested their involvement in both drought and salt stress responses. Interestingly, the ortholog of *PgWRKY62* in rice (*OsWRKY11*) was also shown to be involved in multiple abiotic stresses [49]. Previous studies also showed that individual transcription factors might be involved in multiple signaling pathways. For example, *AtWRKY39* involved in both heat stress and hormone signaling [47]. Interestingly, W-box element (binding site for WRKY TF) was located in upstream region of *PgWRKY62* and *PgWRKY33*, which suggests that these *PgWRKY* genes might auto-regulate their expression during stress conditions. Taken together, *in-silico* analysis of identified PgWRKYs and their transcriptional profiling would help in candidate *PgWRKY* genes selection for delineating their functional roles in abiotic stress tolerance mechanism of pearl millet.

## Conclusions

The research reported in the manuscript describe the genome wide identification of WRKY TFs and their transcriptional profiling in response to dehydration and salinity stress. Furthermore, our findings provide the foundation for further functional characterization and identification of the regulatory mechanism of *PgWRKYs* in plant stress responses. Additionally, candidate *PgWRKYs* can be employed for crop improvement using molecular breeding techniques and genome editing tools to enhance agricultural production for ensuring future food security.

## Methods

### *In-silico* database mining for the identification of WRKY proteins from *P. glaucum*

The protein sequences containing WRKY domain of *Arabidopsis thaliana*, *Oryza sativa* (rice), and *Setaria italica* (foxtail millet) were used as reference sequences for the identification of WRKY proteins in *P. glaucum*. A total of 38,579 proteome sequences of *P. glaucum* were retrieved from pearl millet genome database (http://cegsb.icrisat.org/ipmgsc/) [50] and a total of 282 reference sequences (Additional file 15) were downloaded from the Phytozome v12.1.6, Plant Genomics Resource, The Arabidopsis Information Resource (TAIR) ((https://www.arabidopsis.org/) and Oryzabase; An Integrated Biological and Genome Information Database for Rice [51–53]. The search for WRKY protein sequences in *P. glaucum* was initiated with the construction of HMM profile. The approach uses a Position-Specific Scoring System as well as the Hidden Markov Model to generate a HMM profile using HMMER tool v3.2 with default parameters [54] based on the degree of conservation from multiple sequence alignment. Initially, the reference sequences were aligned using Clustal omega [55] and the HMM profile was build based on the obtained consensus of the aligned sequences. By using the generated WRKY profile, the HMMER search was performed against the proteome database to identify probable homologous WRKY sequences in *P. glaucum*. Furthermore, the HMMER scan was performed to confirm the presence of WRKY domain in identified putative WRKY homologs using the PFAM domain (PF03106) [56].

### Phylogenetic tree construction and sequence analysis

The WRKY protein sequences of *P. glaucum, A. thaliana, O. sativa* and *S. italica* and were aligned using MUSCLE with default parameters [57] and imported into the MEGA v7.0 software [58] to construct an evolutionary relationship tree using Maximum likelihood method. The parameters used for the phylogenetic tree construction are bootstrap test method for 1000 replications; Substitution model: Jones-Taylor-Thronton (JTT) model; Rates among sites: gamma distributed (G) and Gaps/Missing Data Treatment: Partial deletion. Each WRKY protein sequence of *P. glaucum* was analyzed for their physiochemical characteristics such as the number of amino acids, molecular weight, isoelectric point, instability index, aliphatic index and hydropathicity using ProtParam tool on the ExPASy server [59].

### Chromosomal mapping and gene structure

To visualize the chromosomal position of each *WRKY* gene, the chromosomal coordinates with starting and ending points in ascending order were imported into MapInspect v1.0 software (http://mapinspect.software.informer.com/). The physical map of the *WRKY* genes was constructed by locating the genes on each of the seven chromosomes. Coding sequences of identified *WRKYs* were retrieved from pearl millet genome database (http://cegsb.icrisat.org/ipmgsc/) [50] and genomic sequences of each *WRKY* were extracted using available genomic coordinates. The intronic and exonic positions of all *WRKY* genes in *P. glaucum* were predicted using the coding and genomic sequences in GSDS web server v2.0 [60]. Further, these WRKYs were analyzed for conserved motifs using the MEME suite v5.1.0 [61].

### Collinearity mapping and the calculation of synonymous (Ks) & non-synonymous Ka) ratio (Ks/Ka)

*P. glaucum* WRKY sequences were searched for sequence homologs against the WRKY families of *A. thaliana*, *O. sativa* (rice) and *S. italica* (foxtail millet), using BlastP (e-value cut-off 0.001) analysis. The MCScanX v0.8 software [62] was used to identify the orthologous pairs, tandem and segmental duplications among *WRKY* genes. The synteny map of orthologous and paralogous pairs across the species was shown in Circos plot generated by using R software [63]. Further, the substitution rates including the synonymous (Ks) and non-synonymous (Ka) ratio for the potential collinear pairs were estimated using the PAL2NAL server v14.0 [64] by providing pairwise sequence alignment information and the coding sequences of collinear pair. To achieve this, the EMBOSS Water algorithm was used for the pairwise alignment to identify the similar local regions in the collinear pairs and the coding sequence information was downloaded from pearl millet genome database.

### Gene ontology annotation and identification of cis-regulatory elements

The gene ontology (GO) terms for the WRKY proteins were identified using default parameters in Blast2GO v5 tool [65]. Initially, the sequences were screened using BLASTP, followed by mapping, InterProScan analysis and annotation. Furthermore, the biological processes, cellular components and metabolic pathways were predicted using identified GO terms. We have also analyzed the promoter region for identifying the presence of cis-regulatory elements by taking 1500 bp upstream region of all pearl millet *WRKY* genes using the Plant CARE database [66].

### Plants material and stress treatment

Pearl millet germplasm (Preservation Accession number: IP 3757, ISP number: 2347, Collector number: SAR 1148) [67] obtained from Gene bank, International Crops Research Institute for Semi-Arid Tropics (ICRISAT, an Institute of CGIAR with large repository of pearl millet germplasm), Patancheru, India through a material transfer agreement (MTA). Seeds of IP 3757 were sown in pots, filled with a mixture of soil and vermiculture (at a ratio of 1:1, v/v), and then grown in greenhouse conditions having a temperature 25 °C (± 2), photoperiod of 16 h light/ 8 h dark.

The relative expression level of *WRKY* genes were studied in the selected cultivar in different tissues and under drought and salinity stress conditions at different time points. The leaves, stem and root tissues were harvested under normal growth conditions from four-week-old seedlings of pearl millet. Drought condition was created by withdrawing irrigation for 6 days after 4th week of sowing of pearl millet seeds and the control plants were regularly watered on alternate days. On 7th day, irrigation was done for both control and treated plants for their recovery. The leaves of both control and treated plants were collected at day 0, 4, 6 and 8 respectively [68]. For salt stress, four-week-old seedlings were washed and immersed in 250 mM NaCl solution while for control treatment, seedlings were kept in distilled water. Leaf samples were collected from both control and treated plants at time points of 0 h, 3 h (early) and 24 h (late) [29]. At each time point, the leaves of three different plants were harvested as a single sample and immediately frozen in liquid nitrogen and stored at − 80 °C until further analysis.

### Relative expression level of *WRKY* genes

Phylogeny, homologous pairing, sequence similarity and blast results of all WRKY genes of pearl millet were analyzed with well characterized *WRKY* genes of *A. thaliana, O. sativa* and *S. italica* and in response to various abiotic stresses for selection of candidate *WRKYs* genes of pearl millet. The transcript level of these selected WRKYs were analysed to check their differential expression pattern. Primers were designed for selected *WRKYs* using Primer3 input [69] (http://primer3.ut.ee/), according to the following criteria: i) amplicon size of 100–150 bp; ii) primer length of 18–23 bases; iii) melting temperature of 57–63 °C and iv) GC content of 40–60%. The PCR conditions were standardized for each pair of primers using the genomic DNA of pearl millet as a template.

Total RNA was extracted from frozen plant tissues using RNA extraction kit (STRN50, Sigma Aldrich, St. Louis, USA) following the manufacturer’s protocol. RNA concentration and purity were confirmed using Nanodrop 2000 spectrophotometer (Thermo Scientific, Wilmington, DE, USA) for each sample. The integrity of extracted RNA was also ensured by resolving on 1.2% agarose gel containing 18% formaldehyde. For each sample, 1 μg of total RNA was treated with RNase free DNase I (Sigma-AMPD1, St. Louis, USA) and reverse transcribed to synthesize cDNA using first strand cDNA synthesis kit (K1612, Thermo Scientific, MA, USA). Semi-quantitative RT-PCR was done for 35 cycles using prepared cDNA. The qRT-PCR was performed in ABI StepOne Real-Time PCR System (Applied Biosystems, Foster City, CA, USA). The reaction mixtures of 20 μl contained 10 μl of SYBR Premix buffer (Mesa green qPCR master mix-Eurogentec), 1.0 μl from each forward and reverse primer (5 μM), 2 μl (20 ng) of cDNA and 6 μl nuclease free water. Negative control samples were having all the ingredients as mentioned above except the cDNA template. Each reaction was run in three technical and three biological replicates to obtain an average value with respective standard deviation. The amplification conditions were: 95 °C for 10 min followed by 40 cycles of 95 °C for 15 s, 58 °C for 1 min. Fluorescent intensity data was collected, and the reaction specificity was confirmed by melting curve analysis (58 °C to 95 °C, at increments of 0.3 °C). The constitutively expressed *EF1α* [70] and *GAPDH* [71] genes were used as endogenous controls.

## Supplementary information

**Additional file 1: Table S1.** Protein features and chromosomal details of identified 97 PgWRKYs.

**Additional file 2: Table S2.** Different types of WRKY variants in four groups of PgWRKY family.

**Additional file 3: Figure S1.** Group-Wise distribution of *PgWRKY* genes among the seven chromosomes of *P. glaucum.*

**Additional file 4: Table S3.** Motif distribution across all the identified 97 PgWRKYs.

**Additional file 5: Table S4.** List of tandem and segmental duplicated *PgWRKY*s.

**Additional file 6: Table S5.** List of orthologous pair of *P. glaucum* with *S. italica*, *O. sativa* and *A. thaliana*.

**Additional file 7: Table S6.** The Ks/Ka ratio of identified collinear pairs.

**Additional file 8: Table S7.** List of cis-regulatory elements (CREs) present in promoter region of identified *PgWRKYs*.

**Additional file 9: Table S8.** Selected PgWRKYs for expression analysis and their group representation with motif conservation.

**Additional file 10: Table S9.** List of primers used in qRT-PCR analysis for selected *PgWRKYs.*

**Additional file 11: Figure S2.** Standardization of PCR conditions for selected *PgWRKYs* and endogenous control genes (*GAPDH* and *EF1α*) using pearl millet genomic DNA as a template.

**Additional file 12: Figure S3.** Expression level of selected *PgWRKY*s in different tissues (leaves, root and stem) of pearl millet.

**Additional file 13: Figure S4.** Influence of drought stress treatment on four-week old pearl millet seedlings. A) Growth rates of control and treated seedlings at 0th day and 6th day. B) Recovery level on 8th day of drought stressed seedlings after re-watering on 7th day.

**Additional file 14: Figure S5.** Salt stress treatment on four-week-old seedlings of pearl millet (IP3757). A) Control and treated seedlings at 0 h of treatment. B). The response of control and treated seedlings to 250 mM NaCl at 24 h after treatment.

**Additional file 15: Table S10.** A list of accession numbers of reference WRKYs used to access the data analyzed in this study.

## Data Availability

All data analyzed during this study are included in this article and its additional files.

## Abbreviations

ABA: Abscisic Acid
*AtWRKY*: *Arabidopsis thaliana* WRKY
CREs: Cis-regulatory elements
GO: Gene ontology
MeJA: Methyl Jasmonate
*OsWRKY*: *Oryza sativa* WRKY
*PgWRKY*: *Pennisetum glaucum* WRKY
SA: Salicylic acid
*SiWRKY*: *Setaria italica* WRKY
*TaWRKY*: *Triticum aestivum*
TFs: Transcription factors
