# Supplementary figures and images for: Genome-wide identification and expression analysis of WRKY transcription factors in pearl millet (Pennisetum glaucum) under dehydration and salinity stress
Source: BMC Genomics. 2020 Mar 14;21:231. doi: 10.1186/s12864-020-6622-0 (PMC7071642; doi:10.1186/s12864-020-6622-0)

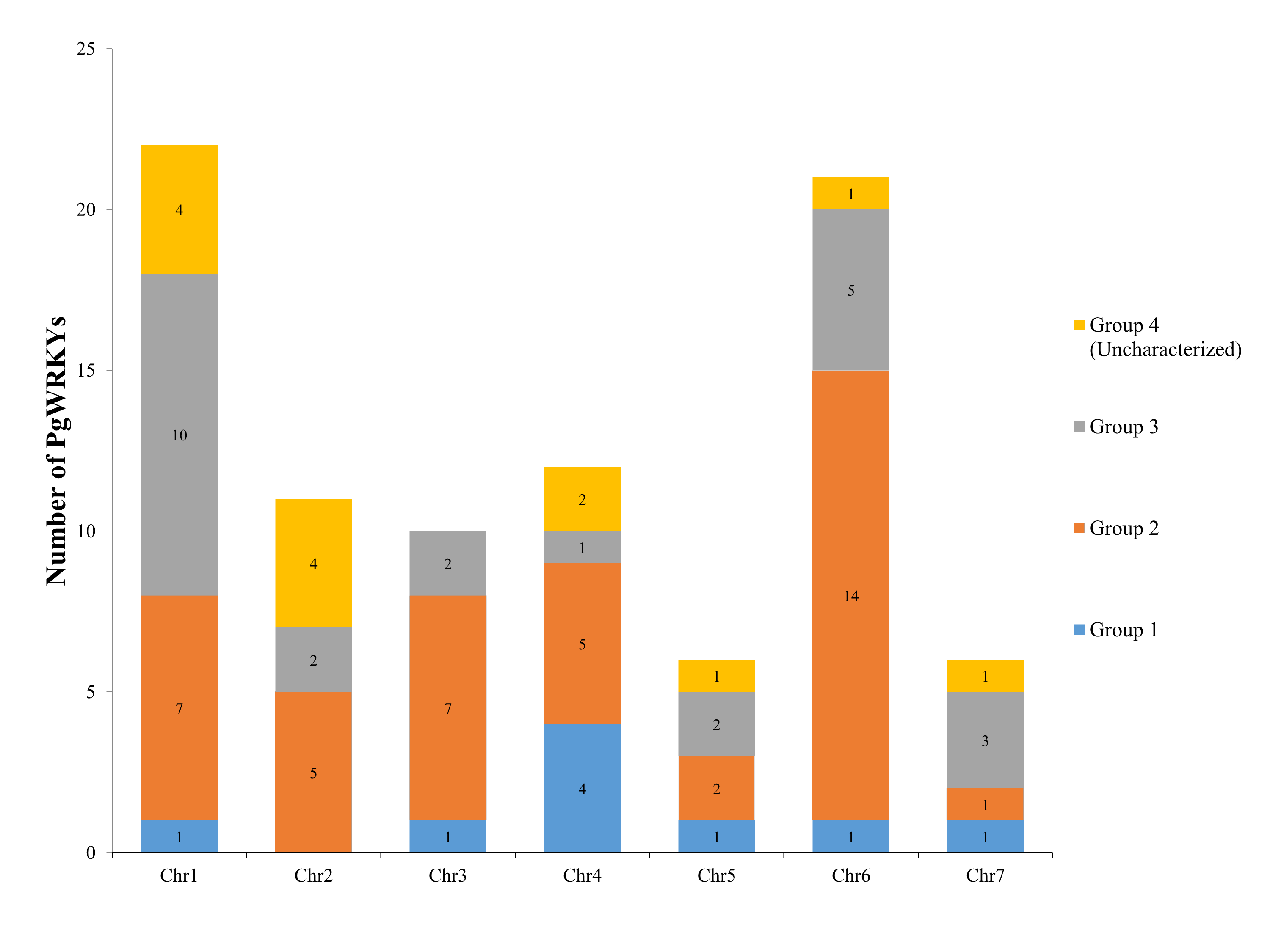

Supplement: Supplementary file 3 — Additional file 3: Figure S1. Group-Wise distribution of PgWRKY genes among the seven chromosomes of P. glaucum. [file 12864_2020_6622_MOESM3_ESM.tif]

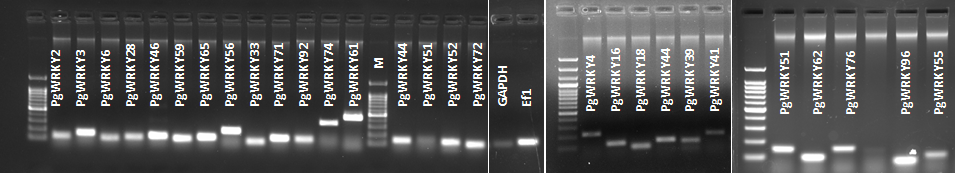

Supplement: Supplementary file 11 — Additional file 11: Figure S2. Standardization of PCR conditions for selected PgWRKYs and endogenous control genes (GAPDH and EF1α) using pearl millet genomic DNA as a template. [file 12864_2020_6622_MOESM11_ESM.tif]

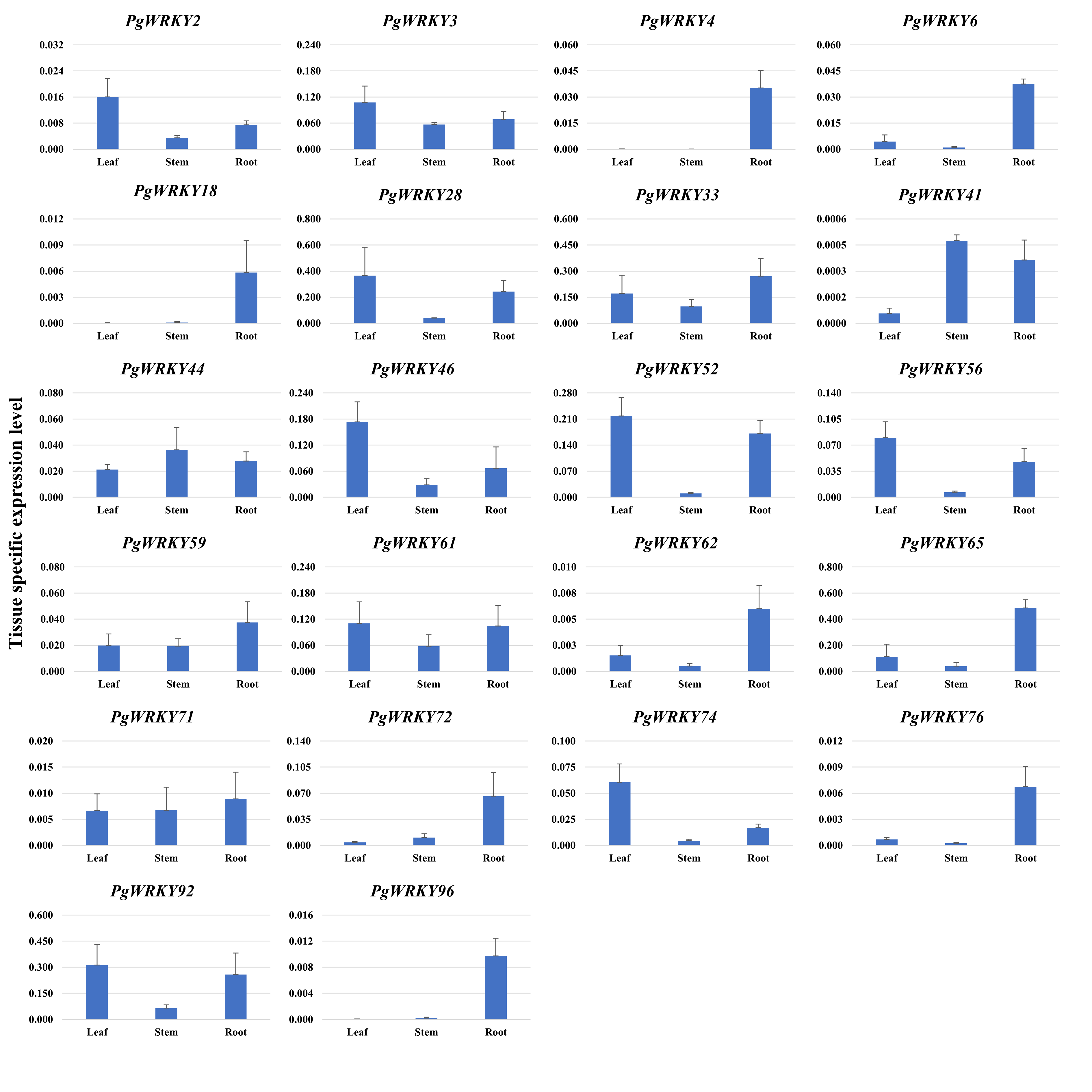

Supplement: Supplementary file 12 — Additional file 12: Figure S3. Expression level of selected PgWRKYs in different tissues (leaves, root and stem) of pearl millet. [file 12864_2020_6622_MOESM12_ESM.tif]

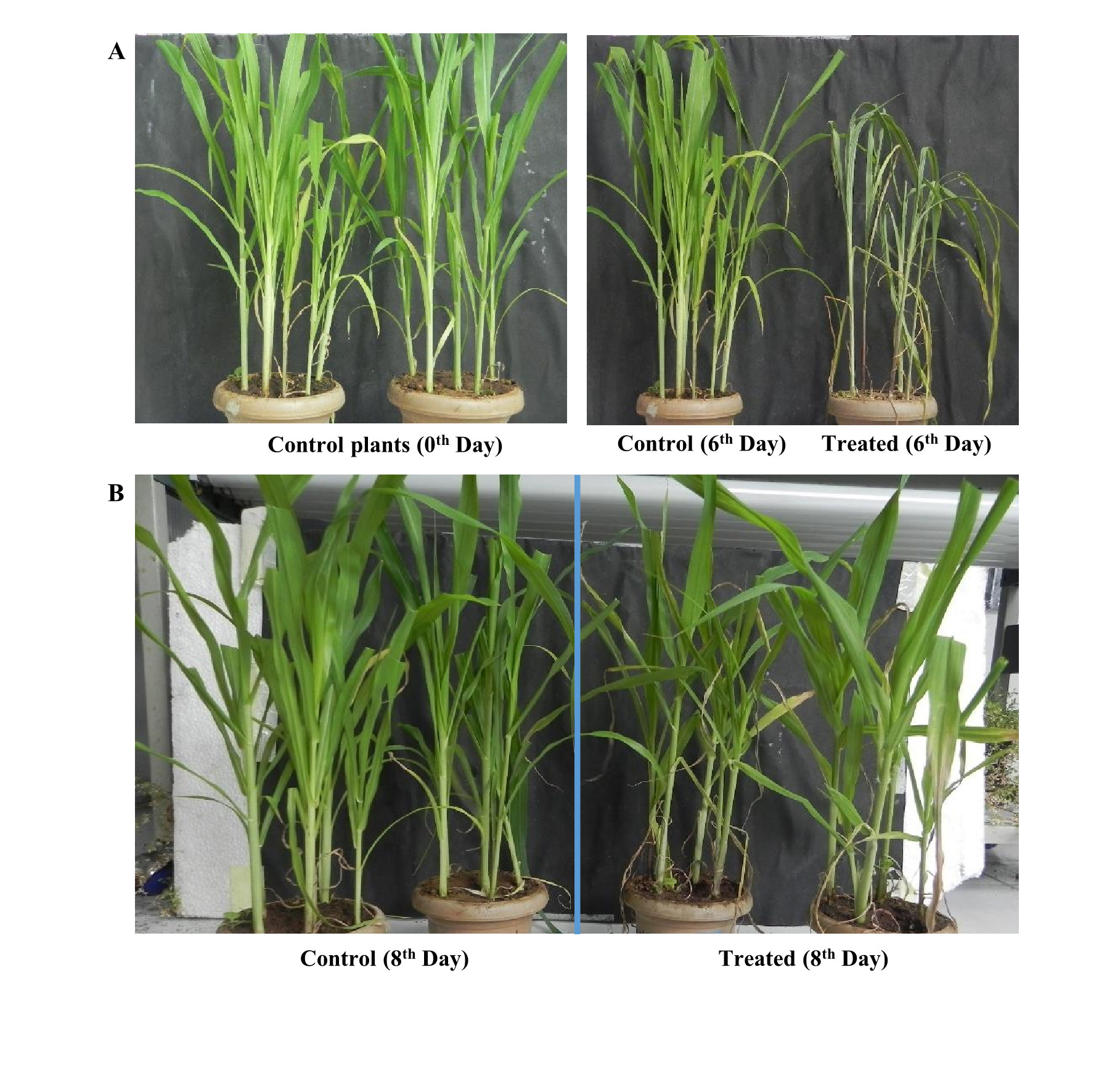

Supplement: Supplementary file 13 — Additional file 13: Figure S4. Influence of drought stress treatment on four-week old pearl millet seedlings. A) Growth rates of control and treated seedlings at 0th day and 6th day. B) Recovery level on 8th day of drought stressed seedlings after re-watering on 7th day. [file 12864_2020_6622_MOESM13_ESM.tif]

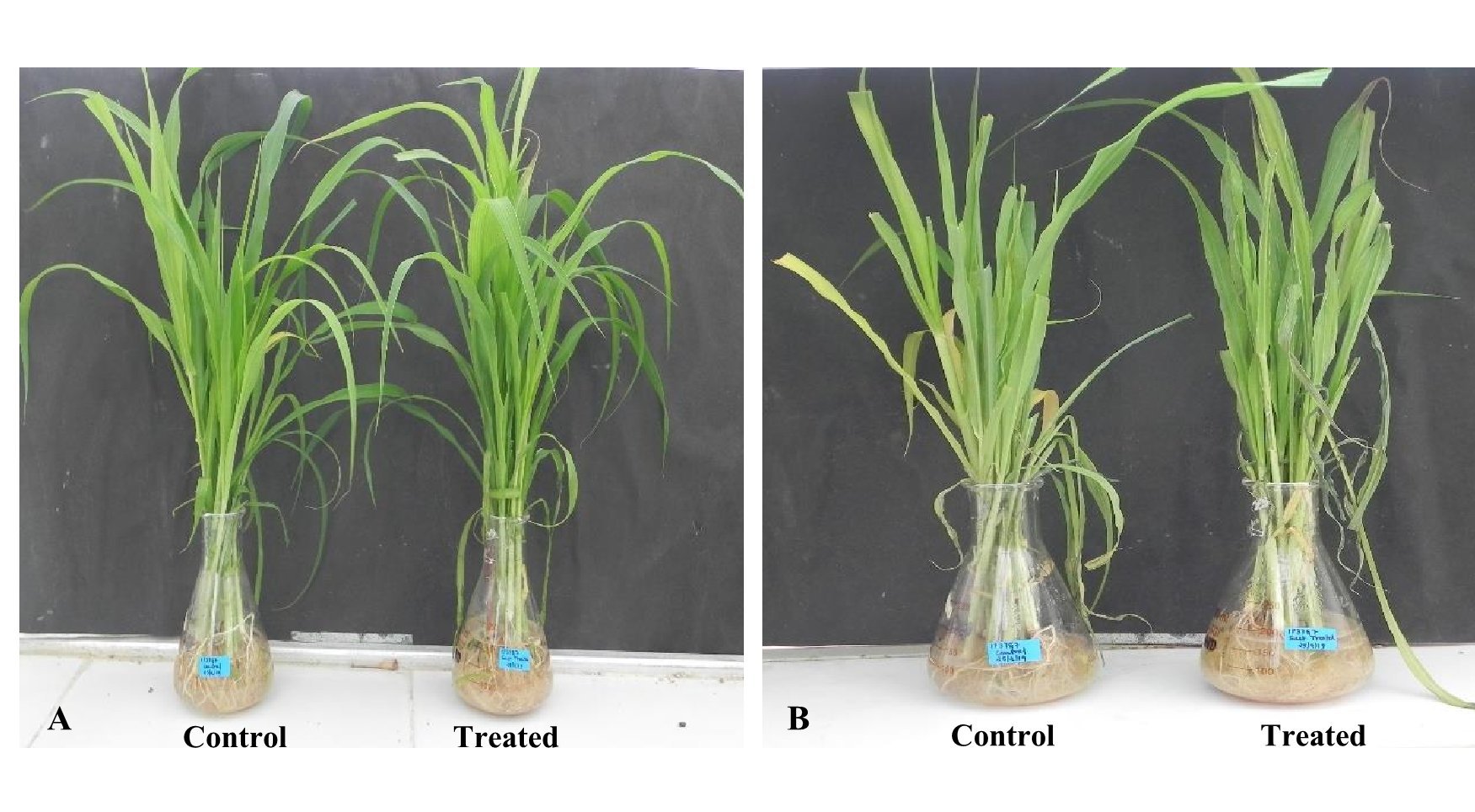

Supplement: Supplementary file 14 — Additional file 14: Figure S5. Salt stress treatment on four-week-old seedlings of pearl millet (IP3757). A) Control and treated seedlings at 0 h of treatment. B). The response of control and treated seedlings to 250 mM NaCl at 24 h after treatment. [file 12864_2020_6622_MOESM14_ESM.tif]
